# From Payload‐First Toward Dual‐Mechanism Antibody–Drug Conjugates

**DOI:** 10.1002/adma.74387

**Published:** 2026-07-30

**Authors:** Xavier Pivot, Sebastian Jung, Sébastien Harlepp, Alexandre Detappe

**Affiliations:** ^1^ Nanomedicine Laboratory Institut Strauss Strasbourg France; ^2^ Equipe labellisée ligue contre le cancer France; ^3^ Strasbourg Drug Discovery and Development Institute (IMS) Strasbourg France; ^4^ INSERM Gustave Roussy Biothérapies Innovantes U1363 Université Paris‐Saclay Villejuif France

**Keywords:** antibody, antibody–drug conjugate, chemistry, conjugate, linker, payload, taxonomy

## Abstract

Antibody–drug conjugates (ADCs) are often described as simple carriers that shuttle cytotoxic payloads to tumors. However, many backbones, exemplified by trastuzumab, are potent biologics whose pharmacology is eroded by conjugation. In this Perspective, we introduce the concept of an antibody exposure deficit. This represents the systematic reduction in both antibody mass and systemic exposure delivered by an ADC relative to its approved unconjugated monoclonal antibody. We show how payload type, drug‐to‐antibody ratio (DAR), linker and payload‐linker hydrophobicity, and conjugation architecture together drive this deficit. Site‐specific DAR 1 formats can mitigate this deficit, unlike higher‐DAR constructs such as antibody–polymer conjugates, where the monoclonal antibody serves primarily as a carrier for effective payloads. This defines a landscape of ADC design to which fragment crystallizable (Fc) activity modulation adds further complexity. Altogether, these strategies span the continuum of possible ADC constructs.

## Graphical abstract

Conjugation converts potent antibodies into underexposed carriers. This perspective defines the antibody exposure deficit and maps a mechanism‐first design space from payload‐first to antibody‐first ADC architectures, integrating DAR, linker chemistry, and Fc engineering to guide rational design of constructs that balance targeted cytotoxicity with preserved antibody pharmacology.

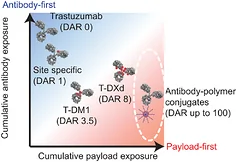

## 1 The Antibody Exposure Deficit: Definition and Clinical Evidence

Antibody–drug conjugates (ADCs) are often introduced through a simplified narrative: the antibody confers specificity, and the cytotoxic payload delivers cell killing [1]. Although intuitive, this framing can obscure a key point for many clinically deployed backbones: the antibody is an effective drug, not only a carrier. This concept is notably illustrated by the management of human epidermal growth factor receptor 2 (HER2)‐positive breast cancer, triggering the revolution of biotherapy in oncology through the clinical development of trastuzumab [2] toward a new generation of trastuzumab‐based ADCs.

Some ADC strategies are increasingly payload‐driven. In HER2‐low tumors, the contribution from trastuzumab is expected to be small, if any, yet trastuzumab deruxtecan (T‐DXd) has demonstrated substantial survival benefit versus physician's choice chemotherapy in metastatic disease [3, 4]. This advantage is likely driven by high‐potency, bystander‐capable payload delivery enabled by a high drug‐to‐antibody ratio (DAR) construct, whereas residual antibody‐mediated HER2 activity at low receptor density mainly serves as a targeting mechanism for payload delivery within the tumor environment.

**FIGURE 1:**
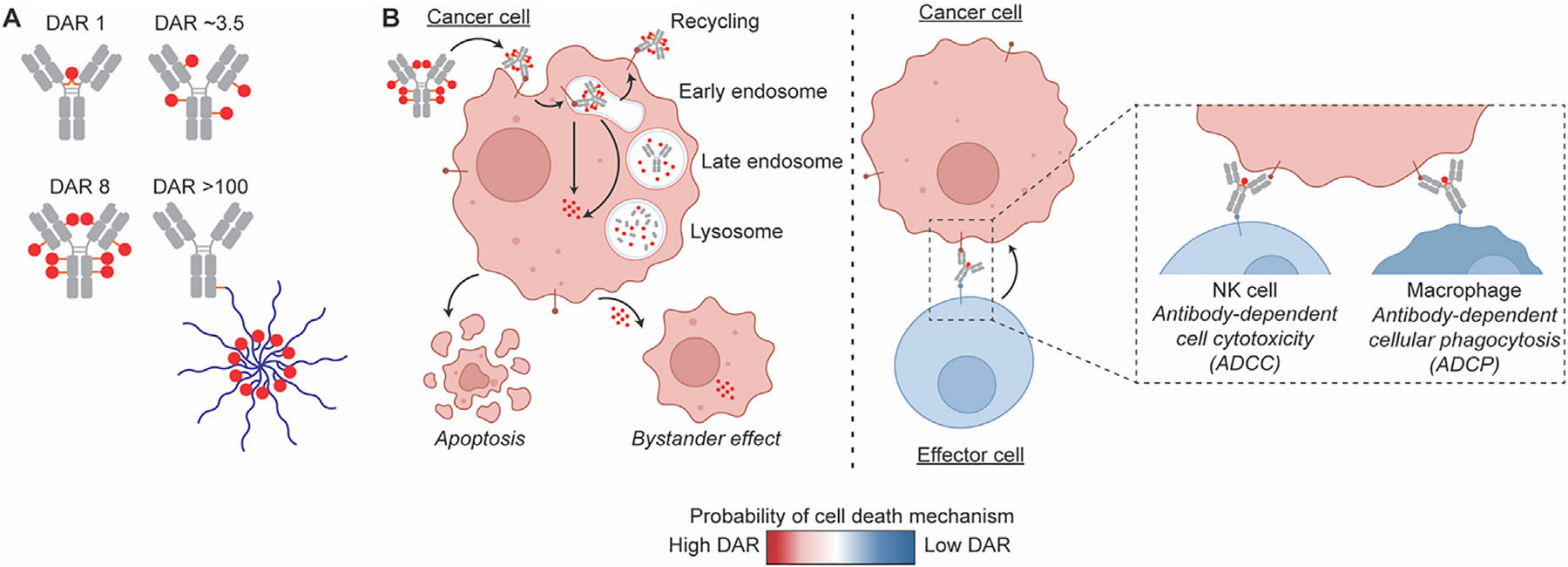
Conjugation architecture governs antibody exposure and the balance between direct cytotoxic and Fc‐mediated immune mechanisms. (A) Representative antibody‐conjugate architectures and their pharmacokinetic consequences, spanning a low‐drug‐to‐antibody‐ratio (DAR) site‐specific conjugate, a conventional stochastic ADC of intermediate DAR, a high‐DAR ADC, and an antibody–polymer conjugate (APC) that carries many payloads on a hydrophilic polymer scaffold. Increasing payload load and surface hydrophobicity progressively accelerate clearance and shorten systemic antibody half‐life, as depicted by the schematic concentration‐time profiles (lower left). (B) The two complementary mechanisms of action available to a conjugated antibody. After binding its target antigen, the conjugate is internalized and trafficked through the endo‐lysosomal pathway, where linker cleavage and proteolytic degradation liberate the cytotoxic payload into the cytoplasm to drive tumor‐cell death, while a fraction of intact antibody escapes degradation and is recycled to the cell surface by the neonatal Fc receptor (FcRn). In parallel, the Fc region recruits innate immune effectors, mediating antibody‐dependent cellular cytotoxicity (ADCC) by natural killer (NK) cells and antibody‐dependent cellular phagocytosis (ADCP) by macrophages. Blue‐to‐red gradients denote the shift from antibody‐driven to payload‐driven activity across conjugate designs, and an orthogonal Fc‐engineering axis (silenced, native, or enhanced Fc) independently tunes the immune‐effector and FcRn‐dependent contributions across all architectures.

**FIGURE 2:**
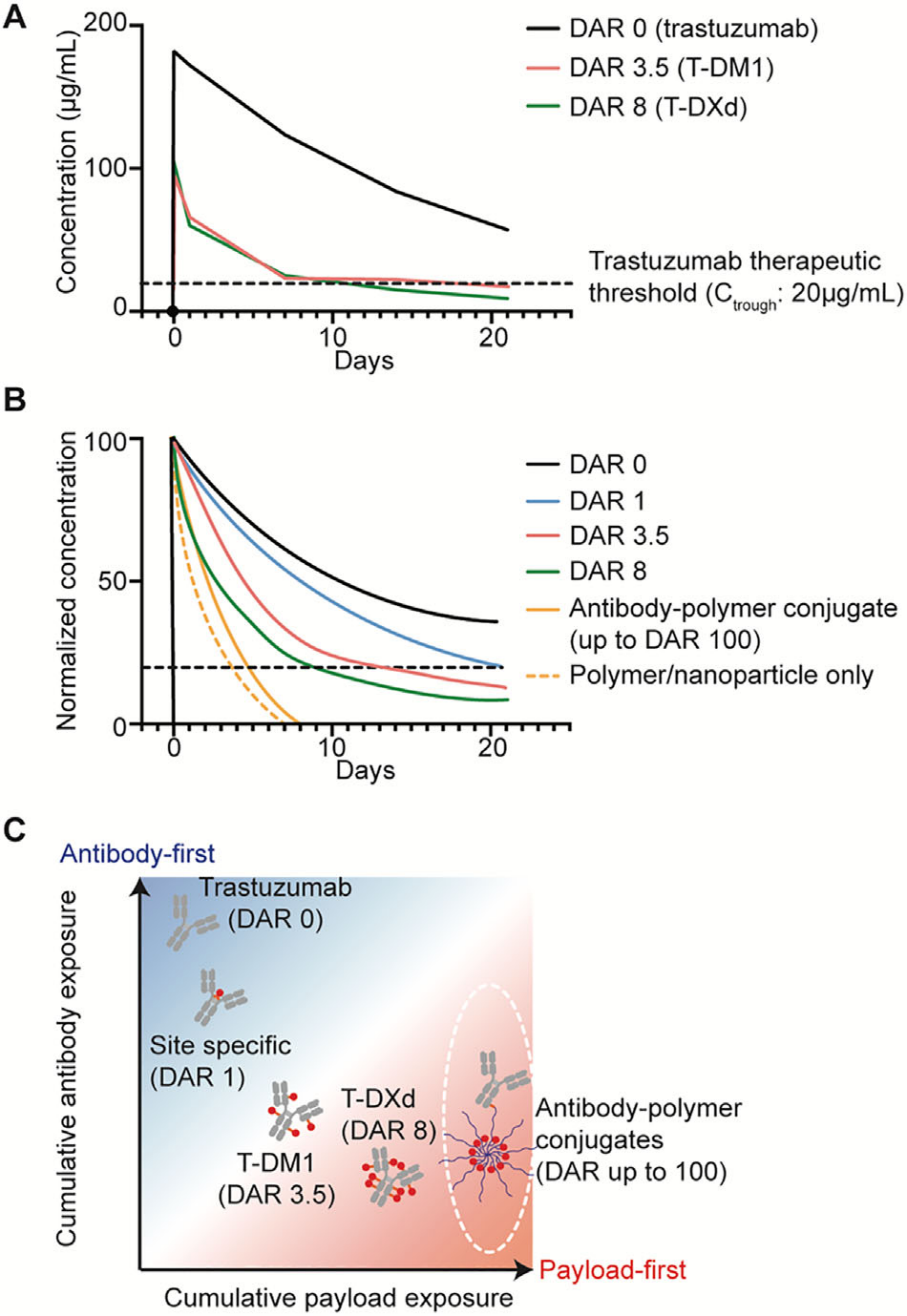
Antibody exposure deficit and mechanism‐first design space for trastuzumab‐based antibody–drug conjugates (ADCs). (A) Illustrative systemic concentration–time profiles for unconjugated trastuzumab (DAR 0, black), T‐DM1 (DAR 3.5, orange), and T‐DXd (DAR 8, green). Trastuzumab efficacy requires maintaining serum concentrations above the biological activity threshold (dashed line, Ctrough ∼20 µg/mL [6, 7]; this threshold is specific to trastuzumab and HER2 and should not be extrapolated to other ADC backbones without independent validation) to sustain HER2 pathway inhibition, receptor downregulation, and Fc‐mediated effector functions. Conjugation reduces functional antibody exposure through lower administered antibody mass and/or accelerated clearance, resulting in trough concentrations that may fall below this threshold. (B) Normalized concentration‐time curves illustrating how increasing DAR and conjugation architecture progressively accelerate antibody disposition. High‐capacity platforms such as antibody–polymer conjugates (APC, up to DAR 100) can substantially increase payload delivery but exhibit markedly shortened systemic half‐life compared with the unconjugated antibody. Polymer/nanoparticle alone (dashed brown line) is shown as a clearance reference. (C) Mechanism‐first design map defined by cumulative payload exposure (x‐axis) and cumulative antibody exposure (y‐axis). Unconjugated trastuzumab (DAR 0) and low‐DAR site‐specific constructs (DAR 1) occupy the antibody‐first region (green), where antibody pharmacology is preserved. Higher‐DAR ADCs (T‐DM1, DAR 3.5; T‐DXd, DAR 8) and APC (DAR up to 100) fall within the payload‐first region (orange), reflecting high payload delivery but reduced systemic antibody exposure. An orthogonal design axis (shown as schematic inset) encompasses Fc activity, which can be silenced, native, or enhanced to independently modulate immune engagement and systemic circulation.

By contrast, in HER2‐positive breast cancer, trastuzumab has been transformative, revolutionizing outcomes in both metastatic and early settings [5]. In this context, a therapeutic contribution from the antibody itself is expected within an ADC construct. For trastuzumab to achieve its full effect, specific pharmacological criteria must be met. These include the recommended intravenous or subcutaneous dosing schedule for trastuzumab, designed to maintain serum concentrations above the biological activity threshold (20 µg/mL [6, 7]) and sustained antibody exposure supporting durable HER2 signaling inhibition, receptor downregulation, and fragment crystallizable (Fc)‐mediated effector functions. When trastuzumab is converted into an ADC, the implicit assumption is often that this antibody biology is retained and that the payload simply adds a second, cytotoxic mechanism (Figure 1). In practice, conjugation frequently changes the delivered antibody exposure and can shift the balance of mechanisms (Figure 2A).

Ado‐trastuzumab emtansine (T‐DM1) is administered at 3.6 mg/kg every 3 weeks, which corresponds to ∼40% less antibody mass per cycle compared with unconjugated trastuzumab [8]. This recommended dose is based on the maximum tolerated dose (MTD) of the ADC, which is driven by toxicity of the payload rather than that of the monoclonal antibody (mAb) [9]. This dose deficit accounts for both the underexposure and the reduced effect of trastuzumab, suggesting the effect is mainly due to the targeted emtansine payload delivery.

Even when the administered antibody mass approaches that of the unconjugated form, as with T‐DXd administered at 5.4 mg/kg (about 90% of the trastuzumab mass), the apparent half‐life is shortened to approximately 7 days compared with ∼28 days for the unconjugated antibody [8]. A major determinant of this pharmacokinetic (PK) alteration is linker hydrophobicity, as the physicochemical properties of the drug‐linker system directly influence conjugate aggregation, nonspecific clearance, and distribution [10]. In the case of T‐DXd, a high average DAR of 8, combined with a relatively hydrophobic drug‐linker system, contributes to this altered pharmacokinetic profile [11]. Despite reduced trastuzumab exposure, T‐DXd retains substantial clinical efficacy due to its topoisomerase I inhibitor payload with bystander activity [12, 13] (Table 1).

**TABLE 1:** Pharmacokinetic comparison of trastuzumab‐based agents at approved regimens.

Parameter	Trastuzumab	T‐DM1	T‐DXd
DAR	0	3.5	∼8
Dose (mg/kg q3w)	6	3.6	5.4
Antibody mass ratio^a^	1.0 (ref)	0.60	0.90
Terminal T½ (days)^b^	∼28	∼4	∼7
PK analyte measured	Total antibody	Conjugate (≥1 DM1)	Intact T‐DXd
Ctrough ss (µg/mL)^c^	∼63	<10	∼11
Dose‐limiting toxicity	Cardiotoxicity	Thrombocytopenia	ILD/pneumonitis

^a^ Antibody mass ratio = administered antibody mass per cycle relative to unconjugated trastuzumab 6 mg/kg [8].

^b^ Terminal half‐life from population PK analyses. Trastuzumab: T½ = 28.5 days, CL = 0.225 L/day, total antibody analyte [14]. T‐DM1: T½ = 3.94 days, CL = 0.676 L/day, conjugate analyte (≥1 DM1) [15]. T‐DXd: T½ ≈7 days, intact T‐DXd analyte [16]. Because these CL and T½ values measure different analytes, they are not directly comparable across agents.

^c^ Ctrough at steady state from EMA EPARs: trastuzumab Cmin, ss ≈63 µg/mL, total antibody (Herceptin EPAR, Section 5.2 [8]); T‐DM1 Ctrough <10 µg/mL, conjugate analyte (Kadcyla EPAR, Section 5.2 [8]); T‐DXd Ctrough ≈11 µg/mL, intact T‐DXd (Enhertu EPAR, Section 5.2 [8]). Because these analytes differ, Ctrough values cannot be directly compared across agents. Formal calculation of the antibody exposure ratio (AER) requires measurement of the same analyte (total antibody) across all constructs, data not yet available in published literature.

Importantly, the dose‐limiting toxicities of trastuzumab‐based ADCs are driven by the payload rather than the antibody. For T‐DM1, thrombocytopenia and hepatotoxicity define the MTD [9], while for T‐DXd, interstitial lung disease (ILD) is the principal safety concern. In both cases, the administered antibody mass is constrained by payload toxicity, not by antibody‐related adverse events. This observation reinforces the concept of an antibody exposure deficit: the antibody dose is effectively capped below the exposure required for optimal antibody pharmacology because the payload reaches its tolerability limit first. The DESTINY‐Breast03 trial, which directly compared T‐DXd and T‐DM1 in HER2‐positive metastatic breast cancer, demonstrated markedly superior progression‐free survival with T‐DXd (median 28.8 vs. 6.8 months; hazard ratio [HR] 0.33), confirming that conjugation design choices, that is, DAR, linker, and payload, translate into dramatically different clinical outcomes even when two ADCs share the same antibody backbone [17, 18]. Notably, the distinct toxicity profiles (ILD for T‐DXd vs. thrombocytopenia for T‐DM1) further illustrate that the payload, not the antibody, defines the therapeutic window and constrains antibody exposure. Population pharmacokinetic analyses have confirmed that T‐DM1 exhibits time‐dependent clearance with exposure decreasing over successive cycles [15], while T‐DXd shows nonlinear disposition consistent with target‐mediated drug disposition [16]. These analyses further support the need to evaluate antibody and payload kinetics separately.

We define this systematic reduction in antibody exposure, relative to the parent mAb, as the “antibody exposure deficit.” Its clinical relevance depends on the role of the antibody backbone: it is most consequential when the parent antibody has intrinsic antitumor activity, requires sustained target engagement, or mediates Fc‐dependent immune recruitment, as exemplified by trastuzumab in HER2‐positive disease. Accordingly, optimizing the DAR, potentially through lower‐DAR site‐specific formats, may help preserve trastuzumab concentrations above the biological activity threshold while maintaining targeted payload delivery (Figure 2B). We therefore propose the antibody exposure ratio (AER), defined as the ratio of total‐antibody area under the curve (AUC) for the ADC to that of the parent antibody at its approved reference regimen: AER = AUC(ADC, total antibody) / AUC(parent mAb, reference regimen). An AER of 1.0 indicates full preservation of antibody exposure, whereas values below 1.0 quantify the magnitude of the deficit. Importantly, AER is a plasma pharmacokinetic descriptor of the antibody component and should not be interpreted as a surrogate for ADC efficacy. It does not directly capture intratumoral antibody or payload exposure, which may diverge because of deconjugation, catabolism, and spatial heterogeneity. Its value is therefore greatest when interpreted alongside pharmacodynamic and tissue‐distribution data, particularly for antibodies with established exposure‐dependent activity such as trastuzumab. Any AER value must be reported with the analyte measured, whether total antibody or conjugated antibody, as well as the pharmacokinetic endpoint used, such as AUC, steady‐state Ctrough, or steady‐state Cmin, because these can diverge as deconjugation and catabolism progressively decouple antibody and payload kinetics. For this reason, currently published pharmacokinetic data for T‐DM1 and T‐DXd do not yet allow a formal cross‐agent AER calculation, since they report different analytes, conjugate for the former and intact ADC for the latter, rather than total antibody (Table 1). This limitation underscores the need for standardized same‐analyte PK reporting across ADC programs. More broadly, total antibody, conjugated antibody, and free payload can diverge over time, so an ADC may deliver adequate payload exposure while underexposing the antibody component, or vice versa, and these scenarios cannot be distinguished when pharmacokinetics is reduced to a single composite metric. The mechanistic relevance of the antibody exposure deficit follows directly from antibody pharmacology: for trastuzumab and other HER2‐targeting antibodies, exposure‐response relationships have been reported, with clearance decreasing and half‐life increasing at higher doses, and low trough concentrations hypothesized to be associated with inferior outcomes, although these relationships remain incompletely characterized. Receptor occupancy, internalization, receptor downregulation, and Fc‐mediated effector functions are exposure‐dependent processes. An ADC that delivers substantially less antibody exposure, as in the case of T‐DM1 compared with trastuzumab, is therefore unlikely to reproduce the full spectrum of antibody‐driven biology, even before accounting for differences in tissue distribution.

Clinical efficacy of an ADC can still be strong if the payload dominates, but mechanistic interpretation becomes ambiguous when antibody exposure is assumed rather than measured. For example, switching from trastuzumab to T‐DM1 in the adjuvant setting after an incomplete neoadjuvant response improves survival [19], yet this does not demonstrate that full antibody biology is preserved within the ADC. In settings where the antibody contribution is expected to matter, the hypothesis that antibody underexposure could influence outcomes should be tested prospectively with pharmacology‐guided analyses rather than inferred from nominal dose alone [20].

The magnitude of the deficit is shaped not only by the payload, but also by the mean DAR and the distribution of DAR species and its time evolution in vivo. Multiple studies have demonstrated that in heterogeneous conjugates, higher‐DAR species are more hydrophobic and clear faster from circulation, a phenomenon well documented for both lysine‐based and cysteine‐based ADCs [21]. Consequently, the apparent average DAR decreases over time as high‐DAR species are depleted. This dynamic further decouples total antibody exposure from payload exposure and complicates cross‐platform comparisons. This differential clearance also affects tumor microenvironment residence time: rapidly cleared high‐DAR species may have reduced opportunity for tumor accumulation and penetration compared with lower‐DAR species that maintain longer circulation [22]. It also reinforces the notion that DAR should be treated as a distribution linked to clearance and tumor residence time, not as a single static number reported at release.

Although trastuzumab illustrates the problem sharply because the parent antibody is clinically active, the same logic applies broadly to other ADC platforms. It is less critical when the antibody functions primarily as a targeting ligand and payload delivery is intentionally dominant. Distinguishing these scenarios early and designing accordingly is essential to avoid building a construct that is mechanistically mismatched to its intended clinical use.

The approach proposed here extends beyond HER2‐targeting ADCs. For sacituzumab govitecan (Trop‐2 [trophoblast cell‐surface antigen 2], DAR ∼7.6), the hydrophilic CL2A linker partially mitigates the pharmacokinetic penalties of high DAR, illustrating how linker chemistry modulates the deficit independently of drug loading [23]. For enfortumab vedotin (Nectin‐4, DAR ∼3.8), the antibody backbone was not developed as a standalone therapeutic, placing the construct firmly in the payload‐first category by design [24]. The historical example of gemtuzumab ozogamicin (CD33 [cluster of differentiation 33], DAR ∼2‐3) provides a cautionary illustration: heterogeneous conjugation with a hydrophobic calicheamicin payload resulted in an unfavorable pharmacokinetics and safety profile, contributing to initial market withdrawal and subsequent reformulation at a lower dose [25]. In each case, the interplay among DAR, linker hydrophobicity, and conjugation homogeneity determines the magnitude of the antibody exposure deficit and, consequently, the mechanistic balance between antibody‐ and payload‐driven activity. The AER metric is most informative when the parent antibody has established clinical activity, as with trastuzumab or rituximab, but it also provides a rational pharmacokinetic descriptor for evaluating whether Fc‐mediated or signaling‐dependent antibody functions are preserved in any ADC construct.

B‐cell maturation antigen (BCMA) targeting follows the same logic: belantamab mafodotin (anti‐BCMA, monomethyl auristatin F payload) is a payload‐dominant ADC in which antitumor activity is attributed to the payload rather than to intrinsic antibody signaling [26]. For payload‐dominant targets such as Trop‐2, BCMA, and Nectin‐4, a large antibody exposure deficit is an expected and acceptable consequence of design rather than a liability, and deliberately maximizing antibody exposure would add cost and chemical complexity without a clear therapeutic rationale. The antibody exposure deficit is therefore a conditional, diagnostic concept: it identifies the settings in which antibody exposure must be preserved, rather than mandating that it be maximized in every ADC.

## 2 Engineering Solutions: From Hydrophilic Linkers to Polymer Architectures

Importantly, the antibody exposure deficit is not an inevitable consequence of conjugating antibodies to payloads. It is the direct result of specific chemical, structural, and materials choices and can therefore be addressed by engineering (Figure 1A). Extensive preclinical studies suggest that simply increasing DAR can enhance in vitro potency but also increase hydrophobicity, aggregation propensity, and nonspecific uptake [27]. Critically, many of these adverse effects are driven by changes in surface hydrophobicity rather than by drug number per se. Hydrophilic linkers and hydrophobicity‐masking segments have therefore been developed to buffer the physicochemical burden imposed by payload loading, relative to conventional high‐DAR ADCs [28]. Polyethylene glycol spacers and polysarcosine units can improve solubility and stability while helping restore antibody‐like pharmacokinetics at higher DAR values [28, 29]. Their success depends on precise tuning of chain length and molecular weight, because spacers that are too short often fail to mask payload hydrophobicity, whereas overly long chains can sterically hinder antigen binding, underscoring the materials engineering challenge at the heart of ADC optimization.

Antibody–polymer conjugates (APC) provide a more radical route to decouple payload loading from antibody surface properties. Instead of directly modifying multiple lysines or cysteines on the antibody, this approach attaches multiple drug molecules to a hydrophilic polymer domain that is linked to the antibody, creating a segregated carrier region that can be tuned independently of the antibody. Bottlebrush polymers exemplify this principle and can support very high effective DAR while maintaining solubility and antigen binding [30]. By sequestering hydrophobic payloads away from the antibody surface, polymer architectures can reduce aggregation and nonspecific uptake relative to conventional high‐DAR ADCs. However, a consistent finding across preclinical studies is that high‐capacity polymer and nanoparticle conjugates exhibit accelerated clearance compared with the unconjugated antibody backbone. A meta‐analysis of antibody‐nanoparticle conjugates demonstrates that pharmacokinetics are driven by the nanoparticle rather than the antibody moiety, regardless of construct design [31]. Recent antibody bottlebrush conjugates similarly show a shortened systemic half‐life compared with the DAR 0 backbone. These observations indicate that high‐capacity shielding mitigates but does not eliminate pharmacokinetic penalties, and that such architectures are best suited to payload‐first applications where maximizing tumor drug delivery takes precedence over preserving antibody exposure [30, 31]. Smaller, stoichiometric APC can partially improve pharmacokinetics versus heavily loaded constructs yet their systemic exposure typically remains reduced relative to the unconjugated antibody [32]. In this regime, the design problem becomes explicitly materials‐driven: balancing payload capacity against clearance, tissue distribution, and neonatal Fc receptor (FcRn)‐dependent recycling.

Beyond pharmacokinetics, APC carry substantial translational risks that temper near‐term expectations. Their chemistry, manufacturing, and controls (CMC) profile is more demanding than that of conventional ADCs: polymer polydispersity, conjugate heterogeneity, and the need for reproducible, scalable synthesis under good manufacturing practice complicate characterization and regulatory qualification [33]. Polymeric and PEGylated carriers can also be immunogenic, eliciting anti‐polymer or anti‐PEG antibodies that drive complement activation and an accelerated blood clearance (ABC) phenomenon on repeat dosing [34]. Finally, like other nanoparticulate agents, these constructs are subject to uptake by the mononuclear phagocyte system (also termed the reticuloendothelial system, RES), with sequestration by hepatic and splenic macrophages contributing to their accelerated clearance [35]. These uncertainties indicate that APC do not, at present, resolve the antibody exposure deficit, and that further fundamental study of their immunogenicity, biodistribution, and manufacturability is warranted before their advantages can be considered established.

## 3 Site‐Specific Low‐DAR Platforms: Toward Antibody‐First Designs

At the opposite end of the design spectrum, site‐specific conjugation platforms now enable the deliberate construction of low‐DAR ADCs, particularly homogeneous DAR 1 formats, which are well defined and compatible with higher antibody dosing [36]. Early work using engineered cysteines, unnatural amino acids, enzymatic tags, and glycoengineering showed that conjugation site and stoichiometry strongly influence stability, clearance, and in vivo performance, and that selected sites can support low‐DAR species with favorable pharmacokinetics [37]. These approaches have matured into platforms that explicitly target DAR 1 constructs. Glycan‐based conjugation, for example, has been extended to produce homogeneous DAR 1 ADCs on native antibodies without extensive re‐engineering. Beyond biological engineering, chemical re‐bridging strategies using octa‐reactive linkers such as divinylpyrimidines can generate homogeneous DAR 1 ADCs from native disulfide bonds and off‐the‐shelf antibodies [38]. Related emerging approaches similarly prioritize minimal antibody manipulation while retaining full control over stoichiometry, reinforcing the trend toward operationally simple DAR 1 ADC platforms. Collectively, these designs can display high serum stability, predictable pharmacokinetics, and potent antitumor activity despite minimal payload loading. Enzymatic transglutaminase‐mediated conjugation to engineered glutamine tags, sortase‐mediated ligation, and cysteine‐based single payload methods similarly enable restricted low‐DAR ADCs with defined sites and stoichiometries [39]. Transitioning toward low‐DAR formats raises a distinct concern, a potential payload dose deficit. Two levers can compensate: ultra‐potent payloads, such as pyrrolobenzodiazepine (PBD) dimers or duocarmycins, can maintain cytotoxicity at low stoichiometry [38, 40], and the improved tolerability can allow higher antibody mass dosing, increasing the cumulative delivered payload while also improving receptor saturation and tissue penetration. Beyond these two primary strategies, several additional approaches can address the payload dose deficit. The improved tolerability of low‐DAR constructs may allow increased dosing frequency (e.g., every two weeks rather than every three weeks), thereby increasing cumulative payload delivery over time. Dual‐payload conjugation strategies, in which two mechanistically complementary payloads are attached to the same antibody, can enhance cytotoxicity per binding event even at low total DAR. Finally, combination regimens pairing low‐DAR ADCs with the unconjugated antibody or immune checkpoint inhibitors could leverage the preserved Fc function of low‐DAR constructs for synergistic immune engagement. Conceptually, these low‐DAR, site‐specific designs prioritize preserving antibody exposure and homogeneity, which is attractive when deep tumor penetration is needed or when the antibody contribution to efficacy is intended to remain substantial.

## 4 A Mechanism‐First Design Space

Polymer‐shielded high‐capacity architectures with very high DAR, such as antibody–polymer conjugates, and low‐DAR site‐specific ADCs, such as homogeneous DAR 1 glycan or cysteine conjugates, define two anchors in a 2D design space with axes balancing antibody exposure and payload activity (Figure 2C). Conventional high‐DAR, hydrophobic ADCs, which often underexpose the antibody relative to the unconjugated backbone, tend to occupy a payload‐first corner where payload exposure is high but antibody exposure is relatively low. High‐capacity polymer architectures push strongly toward higher payload exposure but consistently exhibit accelerated clearance relative to the unconjugated backbone, placing them firmly within the payload‐first region of the design space. Preclinical data have not yet demonstrated polymer‐based architectures that achieve both high payload delivery and preserved antibody‐like pharmacokinetics; accordingly, these platforms are currently best matched to indications where the antibody serves primarily as a targeting moiety rather than as a therapeutically active component [30, 31]. DAR 1 and related low‐DAR, site‐specific constructs occupy an antibody‐first region of high antibody exposure but relatively lower payload exposure. From the standpoint of mechanism, these regions align with payload‐first, dual‐mechanism, and antibody‐first architectures.

This 2D representation naturally extends into a design cube with a third axis, Fc activity, which can be silenced, native, or enhanced through Fc engineering or glycoengineering. Experience with trastuzumab biosimilars underscored that subtle shifts in critical quality attributes, particularly Fc glycosylation, can measurably alter Fc gamma receptor (FcγR) engagement and antibody‐dependent cellular cytotoxicity (ADCC) without obvious changes in antigen binding or antiproliferative activity [41]. Phase III biosimilar trials revealed that batch‐to‐batch variability in fucose and mannose levels of the reference trastuzumab product was associated with differences in pathological complete response that exceeded predefined equivalence margins, and subsequent analyses linked these glycosylation shifts to measurable effects on ADCC and overall survival [42, 43]. Together, these observations highlight that Fc‐mediated mechanisms are sensitive to small structural changes and that minimal antibody alteration can vary with manufacturing. ADC conjugation adds a further perturbation layer by introducing linkers, payloads, and (for stochastic formats) broad distributions of sites and DAR species, increasing the risks of mAb alteration.

Emerging approaches to generate APC often seek a uniform head‐out orientation by coupling through the Fc region. Protocols describe directional conjugation of monoclonal antibodies to polymersome nanoparticles via Fc moieties using metal‐free click chemistry or use the Fc base as a generic anchoring site for nanoparticle attachment [44]. Fc‐based orientation can be appropriate for immunodiagnostics, imaging, or vaccine delivery, where Fc‐mediated effector functions are unnecessary or potentially undesirable. By contrast, it is poorly aligned with antibody‐first or dual‐mechanism ADC concepts in oncology, where Fc gamma receptor engagement, complement activation, and FcRn‐mediated recycling can contribute to efficacy and systemic exposure. In those settings, anchoring via the Fc region can be mechanistically counterproductive because it can impair Fc effector functions and FcRn‐mediated recycling. Hinge‐directed conjugation, Fab‐specific chemistries, and polymer attachment strategies that spare the Fc region are more compatible with preserving both antigen binding and Fc function while enabling control over payload loading and release. From this perspective, the choice between Fc‐based orientation and Fc‐sparing architectures is a mechanistic inflection point that can determine whether materials technologies support or compromise the antibody contribution to the overall therapeutic effect. This mechanistic distinction is supported by studies demonstrating that Fc‐region accessibility directly modulates FcRn binding efficiency and endosomal recycling [45], and that steric occlusion of the Fc domain by conjugation or nanoparticle attachment can measurably reduce effector function and systemic half‐life [31, 46].

## 5 Fc Engineering as an Orthogonal Design Axis

Fc engineering introduces an additional axis in the design space. Structural modifications and glycoengineering can attenuate or amplify Fc gamma receptor binding and complement activation, and Fc variants that prolong half‐life through increased neonatal Fc receptor affinity are already in clinical use [47]. Engineered Fc glycosylation switches and targeted mutations can produce antibodies with minimal Fc gamma receptor binding and reduced ADCC or complement‐dependent cytotoxicity in vitro and in vivo. In parallel, Fc‐silencing mutations such as N297A or LALA [48, 49, 50] and related designs have been deployed to suppress Fc receptor interactions in clinical candidates, while other Fc variants and glycoengineering approaches can enhance binding to activating receptors to boost immune effector recruitment [51]. Together, these technologies provide graded control of Fc biology that can be layered onto ADCs independently of DAR and polymer architecture.

The clinical relevance of Fc engineering in the ADC context is illustrated by several recent developments. Margetuximab, an Fc‐optimized anti‐HER2 antibody with enhanced FcγRIIIa binding, demonstrated improved progression‐free survival compared with trastuzumab in the SOPHIA trial, particularly in patients carrying the low‐affinity FcγRIIIa‐158F allele [52]. Similarly, obinutuzumab, a glycoengineered type II anti‐CD20 antibody with enhanced ADCC through afucosylation, demonstrated superior progression‐free survival compared with rituximab in the CLL11 trial for chronic lymphocytic leukemia [53]. Together, these results establish clinical proof‐of‐concept that Fc engineering—whether through amino acid substitution or glycoengineering—can meaningfully alter therapeutic outcomes. Conversely, FcRn‐binding‐enhancing mutations (e.g., YTE, LS) have been shown to extend antibody half‐life by 2 to 4‐fold in clinical settings [47], raising the question of whether such modifications could offset the accelerated clearance associated with high‐DAR conjugation. This interaction remains largely untested: whether FcRn‐mediated recycling enhancement can quantitatively compensate for the increased nonspecific clearance driven by payload hydrophobicity is an open experimental question. The answer likely depends on the relative contribution of FcRn‐dependent versus FcRn‐independent clearance pathways for a given construct, which in turn is influenced by DAR, linker chemistry, and conjugation site. Furthermore, preserving antibody exposure through low‐DAR or Fc‐optimized designs has direct implications for tumor penetration: sustained receptor saturation and prolonged systemic circulation enable deeper tissue distribution and more uniform intratumoral antibody concentrations, as demonstrated by computational modeling of antibody transport in solid tumors [22].

In a 3D design space, antibody exposure and payload exposure define a plane within which constructs can be classified as payload‐first, dual‐mechanism, or antibody‐first, while Fc activity defines a third axis distinguishing Fc‐silent delivery vehicles from Fc‐competent or Fc‐enhanced therapies. For example, a nanoparticle‐based high‐loading construct with Fc silencing would combine high payload exposure with reduced antibody exposure and low Fc activity, making it suitable for payload‐first applications where the antibody functions primarily as a targeting ligand. A DAR 1 site‐specific ADC with preserved or enhanced Fc function could combine high antibody exposure with controlled payload exposure and high Fc activity, aligning with an antibody‐centric design that exploits both signaling modulation and immune effector mechanisms.

These considerations argue for a more explicit and quantitative strategy for designing and classifying ADCs. Target product profiles should specify not only the intended payload dose and tumor‐to‐normal tissue ratios, but also the minimal antibody exposure deficit and the desired Fc activity profile for the chosen backbone [54, 55].

The choice between Fc silencing and Fc potentiation should be made per indication and per mechanism rather than as a generic default. Fc‐competent or Fc‐enhanced backbones are rational when the antibody contributes through immune effector mechanisms, with HER2‐positive breast cancer (margetuximab) and CD20‐positive lymphoid malignancies (obinutuzumab) being the clearest examples. Fc silencing, by contrast, is most appropriate when Fc engagement adds toxicity without therapeutic benefit [56]. A concrete illustration is the thrombocytopenia associated with T‐DM1: megakaryocytes can internalize the conjugate in a HER2‐independent, FcγRIIa‐dependent manner, releasing intracellular DM1 and impairing megakaryocyte differentiation, which provides a mechanistic rationale for Fc silencing in payload‐first constructs to limit on‐target, off‐tumor uptake [56, 57]. This rationale is, however, construct‐dependent and not universal: for other ADCs, megakaryocyte uptake proceeds largely through FcγR‐independent macropinocytosis, and blocking the FcγRIIa interaction does not abolish the toxicity [14, 56]. Fc design should therefore be matched to the dominant uptake pathway and the intended role of the antibody for each specific indication.

## 6 Unresolved Challenges and Future Directions

Several critical questions remain to be addressed before the antibody exposure deficit concept can be fully operationalized in ADC development.

First, current clinical trial designs do not allow mechanistic deconvolution of antibody versus payload contributions. In any trial in which an ADC based on a clinically active antibody is administered at anantibody exposure different from that of the unconjugated backbone, it remains difficult to determine whether therapeutic effects derive primarily from payload delivery, from residual antibody biology, or from their interaction. Future trial designs should incorporate comparator arms and pharmacology‐guided analyses that explicitly test whether preserving antibody exposure improves outcomes.

Second, the interaction between Fc engineering and conjugation chemistry is largely unexplored. Whether FcRn‐enhancing mutations can offset the accelerated clearance induced by high DAR or hydrophobic linkers remains an open experimental question. Systematic preclinical studies comparing FcRn‐enhanced versus wild‐type backbones across a range of DAR values and linker chemistries are needed to address this gap.

Third, emerging bioorthogonal conjugation strategies, including click chemistry and enzymatic ligation, may enable modular, site‐selective DAR control on clinical‐grade antibodies without genetic re‐engineering, potentially facilitating rapid iteration across the design space described here [38, 39].

Fourth, computational pharmacokinetic and pharmacodynamic modeling, including physiologically‐based pharmacokinetic (PBPK) models, offers the potential to predict antibody exposure deficits before clinical evaluation. Recent advances in mechanistic PBPK modeling of ADC disposition suggest that in silico tools could be used to screen conjugation architectures for their impact on antibody exposure prior to synthesis [58].

Finally, standardized reporting of ADC pharmacokinetics is essential. We propose that preclinical and clinical studies should systematically report total antibody, conjugated antibody, and free payload concentrations as distinct analytes, together with the AER. This metric would enable cross‐platform comparison and rational classification of constructs along the axis proposed here [54, 55].

## 7 Conclusion

The continued evolution of ADCs depends on recognizing the antibody as a pharmacological entity whose exposure and effector functions must be deliberately designed rather than assumed. Addressing the antibody exposure deficit is central to this effort.

Two opposing strategies emerge along the continuum of possible ADC constructs. At one extreme, highly conjugated ADCs are characterized by substantial mAb exposure deficits, with antitumor activity driven predominantly by the cytotoxic payloads, while the antibody essentially serves as a delivery carrier. At the other extreme, DAR 1 ADCs exhibit minimal mAb exposure deficits, allowing for a dual mechanism of action arising from both the antibody and the payload. These strategies are further modulated by modifications of the Fc region and its associated functions, as well as by the physicochemical properties of the linker and/or payload, which in turn alter antibody exposure. Together, these parameters add further complexity to the spectrum of possible constructs.

## Conflicts of Interest

A.D. is co‐founder and CSO of Recobia Therapeutics, which develops subcutaneous delivery platforms for antibody–drug conjugates, and co‐founder of TherO2. X.P. is co‐founder of Recobia Therapeutics and TherO2. S.J. and S.H. declare no conflicts of interest.

## Data Availability

This article is a perspective and does not contain original experimental data. All data discussed are derived from published studies cited in the reference list.
